# Supplementary material for: Role of Short- and Long-Lived Reactive Species on the Selectivity and Anti-Cancer Action of Plasma Treatment In Vitro
Source: Cancers (Basel). 2021 Feb 4;13(4):615. doi: 10.3390/cancers13040615 (PMC7913865; doi:10.3390/cancers13040615)
Supplement: Supplementary file 1 [file cancers-13-00615-s001.pdf]

Supplementary Material

Supplementary Figure S1: Combined effect of RONS and pH in promoting cancer cell death.

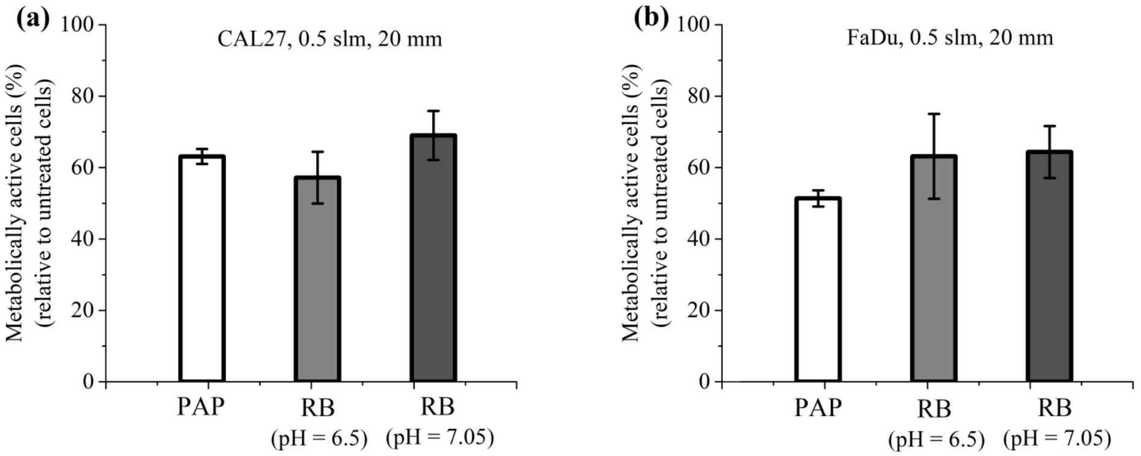

Figure S1. Effect of pH and RONS on the cell viability of CAL27 (a) and FaDu (b) cell lines. Cell viability was assessed 24 h after 1 h of incubation in PAP, in reconstituted buffer (RB) pH 7.05 or in RB pH 6.75. RB consists of 0.17 mM H_2O_2, 0.51 mM NO_2^- and 0.34 mM NO_3^-. The results presented here correspond to a gas flow rate of 0.5 slm (99.8% He / 0.2% O_2), a treatment distance of 20 mm and a treatment time of 12 min. The data are the mean ± SD of 3 independent experiments.

Supplementary Figure S2: Stability of RONS in PAP after storage for several hours at room temperature.

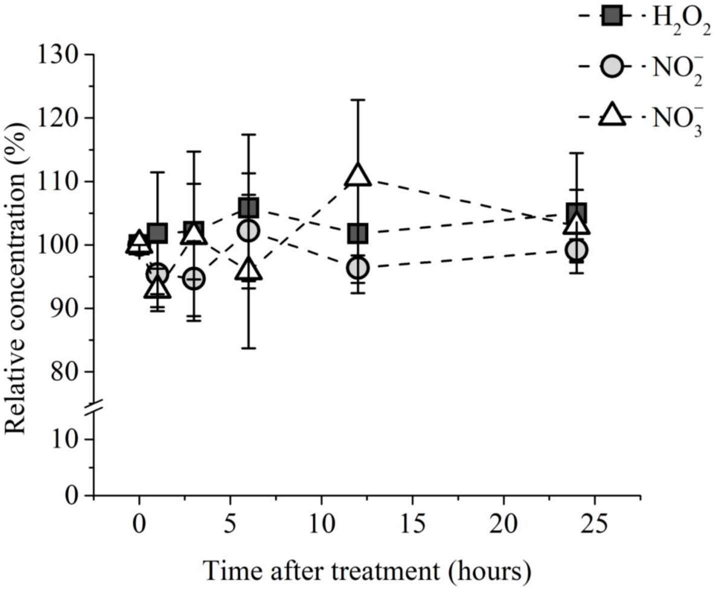

Figure S2. Stability of RONS in PAP over storage time at 23 °C (room temperature). The relative concentration of H_2O_2, NO_2^- and NO_3^- is calculated in relation to their initial concentrations measured immediately after the plasma treatment. The data are the mean ± SD of 2 independent experiments.

Supplementary Figure S3: Lipid peroxidation in CAL27 and FaDu in response to plasma treatment or reconstituted buffer.

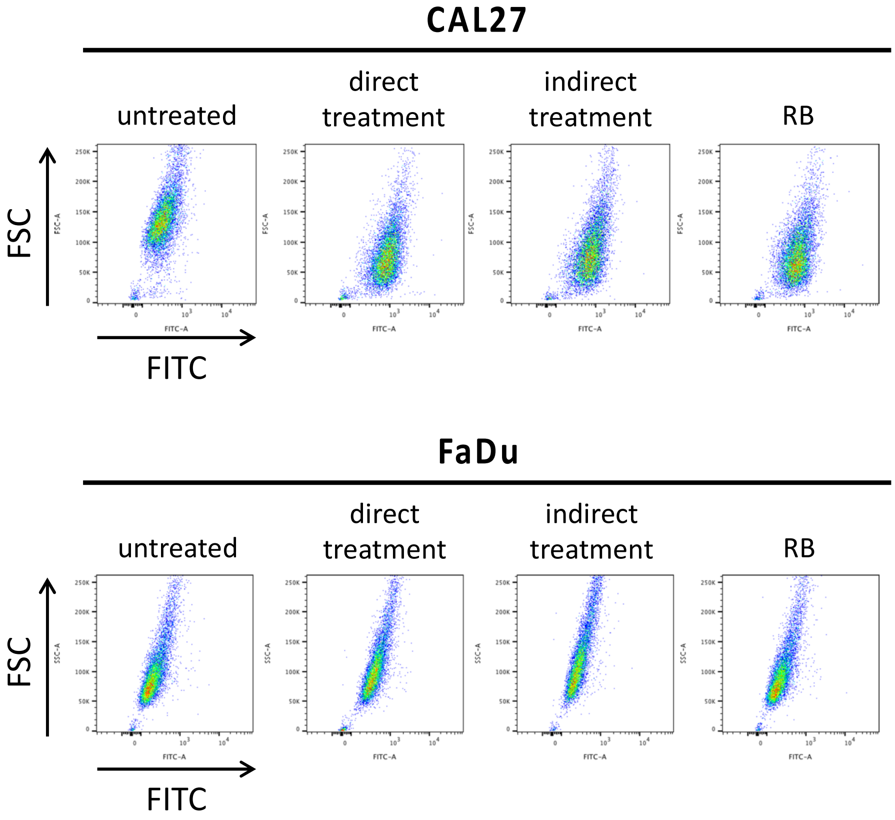

Figure S3. Oxidation of BODIPY 581/591 C-11 after treatment of CAL27 and FaDu cells. The cells were incubated for ½ h with 5 μM BODIPY 581/591 C-11, after which the cells were left untreated or treated in PBS by direct and indirect plasma treatments or reconstituted buffer (RB). After 1 h incubation in untreated or treated PBS, the cells were further incubated for 5 to 6 h in complete cell culture medium, and then collected for FACS analysis. Cells were analyzed by flow cytometry (BD LSRFortessa™ X-20, BD), as per manufacturer instructions. Shown is a representative experiment of the forward scatter versus fluorescence (FITC) for CAL27 and FaDu.

Supplementary Figure S4: Human gingival keratinocytes are very sensitive to direct plasma treatment.

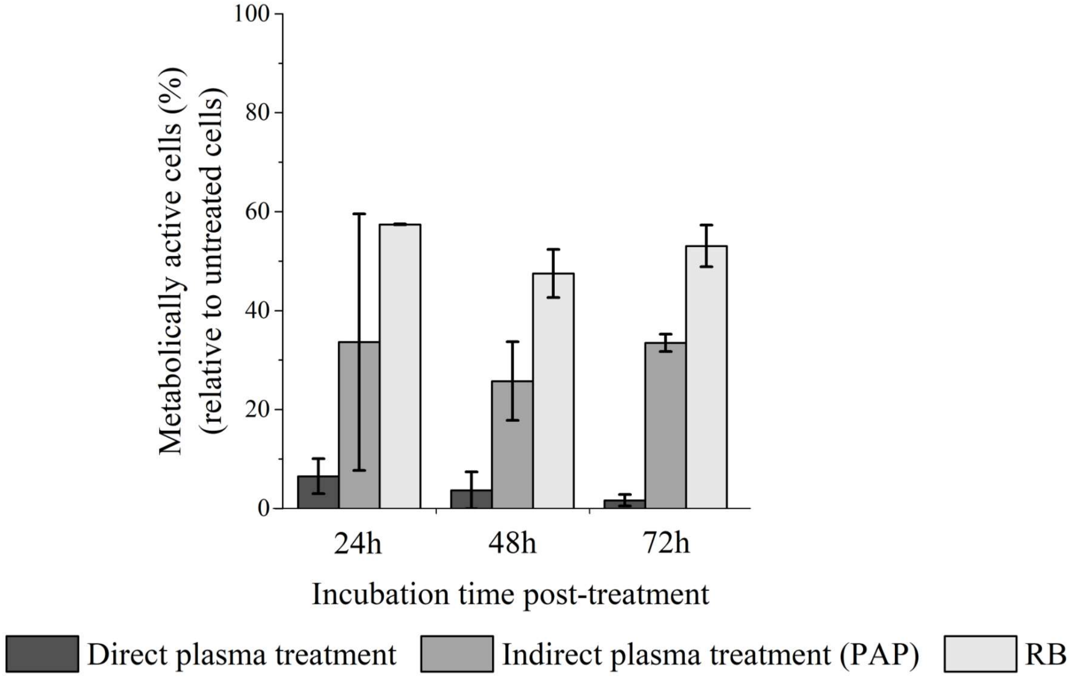

Figure S4. Primary human keratinocytes were exposed to direct plasma treatment, indirect plasma treatment (PAP) and reconstituted buffer (RB). At 24 h, 48 h, and 72 h post-treatment, cell viability was quantified using CellTiter-Glo® luminescent assay (Promega). Conditions used were those corresponding to a gas flow rate of 1 slm (99.8% He / 0.2% O_2), a treatment distance of 8 mm and a treatment time of 12 min. The data are the mean ± SD of 2 independent experiments.

Supplementary Figure S5: Direct plasma treatment triggers cell death in RPE-hTERT cells few hours after the treatment.

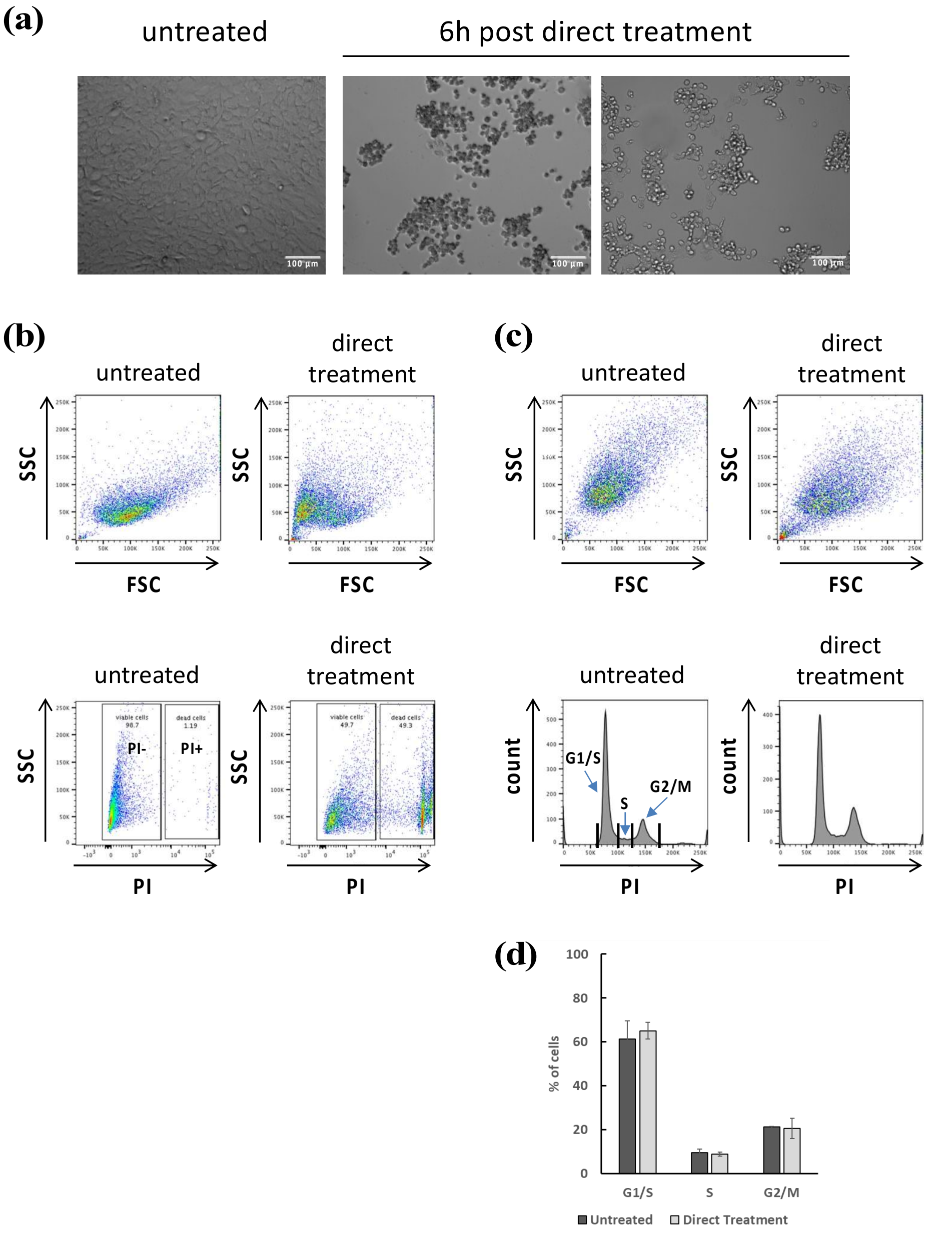

Figure S5. Cell death induced in RPE-hTERT cells by direct plasma treatment. RPE-hTERT cells were exposed to plasma treatment for 12 min and further incubated for 1 h in treated PBS (PAP). After removal of PAP, cells were incubated in complete cell culture medium for 6 h. (a) Photographs of untreated and treated cells were taken 6 h after direct plasma treatment. Photographs were recorded on a Celena® S digital imaging system (Logos biosystem, France) at 10x magnification and are representative of 4 independent experiments. Note that the rounded cells are floating. Thereafter, all cells were collected and analyzed by flow cytometry. (b) Living and dead cells were stained with propidium iodide

(PI). Dead cells (PI positive) represent 39 ± 10 % of the total cells (n = 4). Note that with this technic, cells that were lysed due to plasma treatment are not detected. (c) Cells were fixed in 70 % EtOH and stained with PI. (d) Quantification of cells in G1/S, S, and G2/M of the cell cycle 6 h after direct plasma treatment (n = 4). Conditions used were those corresponding to a gas flow rate of 1 slm (99.8% He / 0.2% O_2) and a treatment distance of 8 mm.

Supplementary Table S1: H_2O_2 and NO_2^- act synergistically in inducing cancer cells death.

(a) CAL27, N = 4

NO_2^- [mM]	H_2O_2 [mM] 0.8	1.6
0.05	**6**	**6**
0.1	**13**	**9**
0.2	**16**	**17**
0.4	**18**	**23**
0.8	**23**	**27**
1.6	**28**	**33**
3.2	**36**	**43**

(b) FaDu, N = 3

NO_2^- [mM]	H_2O_2 [mM] 0.8	1.6
0.05	**5**	**3**
0.1	**9**	**5**
0.2	**8**	**9**
0.4	**14**	**12**
0.8	**16**	**17**
1.6	**22**	**23**
3.2	**32**	**27**

Antagonism / Synergy

Table S1. H_2O_2 and NO_2^- act synergistically in inducing cancer cells death. Synergy analysis for a) CAL27 and b) FaDu. Identical results were obtained with all three models used (Loewe, Bliss and HAS). The numbers in bold correspond to the synergy/antagonism score for each dose combination of H_2O_2 and NO_2^-. The number of independent experiments implemented for this analysis is reported in the top left corner as N.
